# Gambogenic Acid Kills Lung Cancer Cells through Aberrant Autophagy

**DOI:** 10.1371/journal.pone.0083604

**Published:** 2014-01-10

**Authors:** Wang Mei, Chen Dong, Cheng Hui, Li Bin, Yan Fenggen, Su Jingjing, Peng Cheng, Sun Meiling, Hu Yawen, Wang Xiaoshan, Wang Guanghui, Chen Zhiwu, Li Qinglin

**Affiliations:** 1 Department of Pharmacology, Anhui Medical University, Hefei, Anhui, People's Republic of China; 2 Key Laboratory of Xin'an Medicine, Ministry of Education, Anhui University Of Traditional Chinese Medicine, Hefei, Anhui, People's Republic of China; 3 Laboratory of Molecular Neuropathology, Department of Pharmacology, Soochow University College of Pharmaceutical Sciences, Suzhou, Jiangsu, People's Republic of China; University of Quebec at Trois-Rivieres, Canada

## Abstract

Lung cancer is one of the most common types of cancer and causes 1.38 million deaths annually, as of 2008 worldwide. Identifying natural anti-lung cancer agents has become very important. Gambogenic acid (GNA) is one of the active compounds of Gamboge, a traditional medicine that was used as a drastic purgative, emetic, or vermifuge for treating tapeworm. Recently, increasing evidence has indicated that GNA exerts promising anti-tumor effects; however, the underlying mechanism remains unclear. In the present paper, we found that GNA could induce the formation of vacuoles, which was linked with autophagy in A549 and HeLa cells. Further studies revealed that GNA triggers the initiation of autophagy based on the results of MDC staining, AO staining, accumulation of LC3 II, activation of Beclin 1 and phosphorylation of P70S6K. However, degradation of p62 was disrupted and free GFP could not be released in GNA treated cells, which indicated a block in the autophagy flux. Further studies demonstrated that GNA blocks the fusion between autophagosomes and lysosomes by inhibiting acidification in lysosomes. This dysfunctional autophagy plays a pro-death role in GNA-treated cells by activating p53, Bax and cleaved caspase-3 while decreasing Bcl-2. Beclin 1 knockdown greatly decreased GNA-induced cell death and the effects on p53, Bax, cleaved caspase-3 and Bcl-2. Similar results were obtained using a xenograft model. Our findings show, for the first time, that GNA can cause aberrant autophagy to induce cell death and may suggest the potential application of GNA as a tool or viable drug in anticancer therapies.

## Introduction

Lung cancer has been one of the most common types of cancer for several decades and accounts for 15–20% of all cancer-related deaths globally [1]–[2]. By 2008, an estimated 1.61 million new cases per year were reported worldwide. Lung cancer is a major cause of death in the developed world and the most common cancer in China [3]. Surgical resection is the primary method of treatment for lung cancer. However, chemotherapy/radiation therapy is still the effective treatment for patients with advanced non-small cell lung cancer (NSCLC) or small cell lung cancer [4]. Consequently, novel therapeutic strategies and drugs are urgently required for the treatment of lung cancer.

Autophagy is a physiological self-digestive process that degrades cytoplasmic components to sustain cellular metabolism during nutrient deprivation and/or metabolic stress. During autophagy, macromolecules, long-lived proteins and damaged organelles (such as the endoplasmic reticulum and mitochondria) are surrounded by autophagosomes. The autophagosomes then fuse with lysosomes, where the sequestered contents undergo degradation and recycling by resident hydrolases. Autophagy is important in all cells for the removal of long-lived proteins or damaged organelles. This capacity causes autophagy to be a promising candidate for a survival mechanism in response to several stresses [5]. However, several recent studies have suggested that autophagy also functions as a pro-death mechanism caused by anti-tumor therapy [6]–[9]. Indeed, autophagic cell death is considered to be programmed cell death type II, whereas apoptosis is programmed cell death type I [10]. These two types of cell death have been described as distinct forms of cell death; however, many studies show cross-talk between the two types. For example, p53, which is a potent inducer of apoptosis, can also induce autophagy through increasing the expression of *dram*, a direct p53 target gene [11]. Beclin 1/Atg 6 is part of a Type III PI3 kinase complex that is required for the formation of the autophagic vesicles. Interference with Beclin 1 can prevent the induction of autophagy. Disruption of the interaction between the BH3 domain of Beclin 1and Bcl-2 leads to increased autophagy [12]–[13].

**Figure 1:**
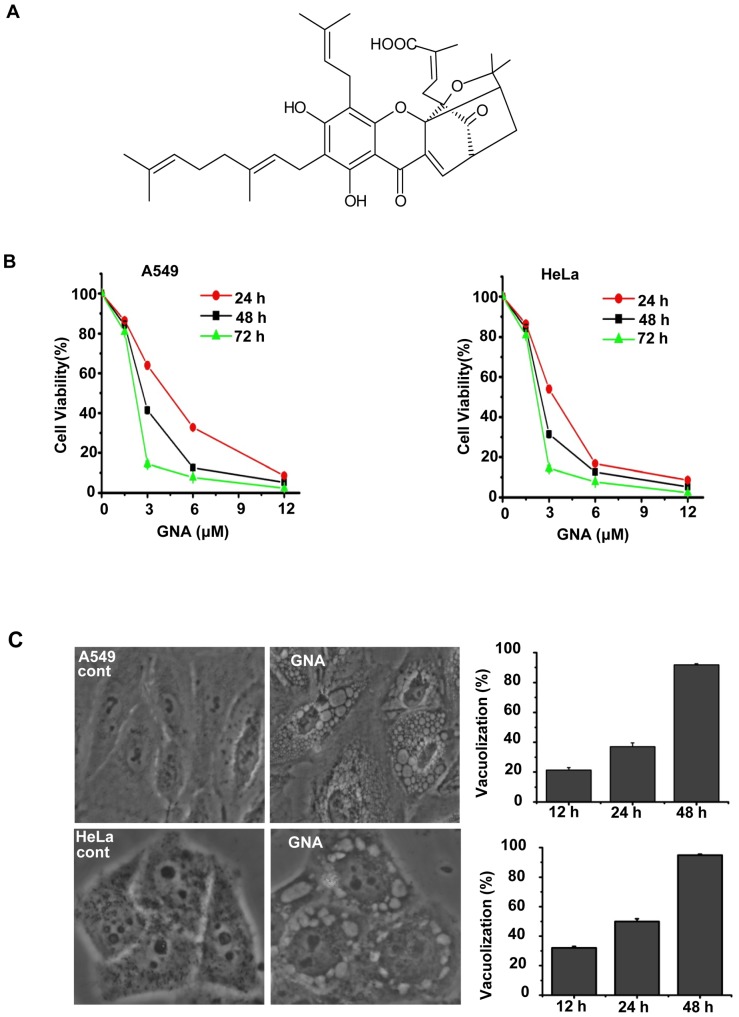
GNA inhibits growth and induces cell death in cancer cells. A, The chemical structure of GNA. B, GNA inhibits growth and inhibits cell death in cancer cells. A549 and HeLa cells were treated with various concentrations of GNA for the indicated periods of time, and cell proliferation was analyzed by the MTT assay. C, Untreated cells (control) or cells treated with 3 µM GNA for 24 hours were directly observed under a phase contrast microscope (left) and cell vacuolization was quantitated (right); at least 600 cells were observed. Means ± SEM, n = 3.

Gambogenic acid (GNA, C38H46O8, MW 631.32) (Figure 1A) is one of the main active components of Gamboge, a resin exuded from the Garcinia hanburyi tree [14]. Gamboge is a traditional medicine that was used as a drastic purgative, emetic, and vermifuge for treating tapeworm. Gamboge has been used in the treatment of cancer, including breast cancer, lung cancer, lymphatic sarcoma and carcinoma cutaneum [15], in a clinic in China and has shown exciting effects [16]. Recently, accumulating evidence has demonstrated that the main active component GNA has a wider spectrum of anti-tumor effects and lower toxicity [17]–[19]. The molecular and biochemical effects of GNA include the induction of apoptosis in various cancer cell lines and animal models of carcinogenesis [20]–[24]. Yu et al. have found that GNA can cause GSK3b-dependent G1 arrest in lung cancer cells and suggested that GNA may induce cell death through autophagy rather than apoptosis [25]. However, the molecular mechanisms underlying the effects of GNA remain unclear. The present studies reveal the molecular mechanism and role of autophagy in GNA-induced tumor cell death.

## Materials and Methods

Mice were maintained in a specific pathogen-free environment. All animal experiments were approved by the animal welfare advisory committee of the University of Science and Technology of China (Permit Number: 2010-11). Adequate measures were taken to minimize pain and discomfort in compliance with national regulations.

### 1. Reagents and antibodies

Gambogenic acid (GNA) (Figure 1A), isolated from Gamboge, was supplied by Prof. Xiaoshan Wang's lab. The GNA purity was 99%, which was determined by HPLC-DAD. MTT, monodansylcadaverine (MDC), propidium iodide (PI), Annexin V, acridine orange and Hoechst 33342 were purchased from Sigma. Rapamycin and trehalose were kind gifts from Prof. Guanghui Wang. MitoTracker Red, LysoTracker Red and LysoSensor Green DND-189 were obtained from Invitrogen. LC3 antibodies were purchased from Novus (NB100-2220), p62 antibodies were from Biomol (PW9860), P70S6K (1494-1), p-P70S6K (3204-1) and p-p38 (1229-1) antibodies were from Epitomics. GAPDH antibody was purchased from CHEMICON (MAB374). GFP (sc-9996), caspase-3 (sc-7148), p53 (sc-6243), Bax (sc-7480), Beclin 1 (sc-11427), and Bcl-2 (sc-7382) antibodies were purchased from Santa Cruz Biotechnology. The reagents were dissolved in phosphate-buffered saline (PBS), except GNA and rapamycin, which were prepared in DMSO.

### 2. Cell culture

The human lung adenocarcinoma cell line A549 was purchased from the Cell Bank of Shanghai Institute of Cell Biology. The human epithelial carcinoma cell line HeLa and established GFP-LC3/HeLa cells were supplied by Prof. Guanghui Wang [26]. For the establishment of a stable cell line expressing EGFP-fused LC3, Hela cells were transfected with EGFP-LC3, and individual clones stably expressing GFP-LC3 were selected using 0.2 mg/mL G418. One moderate expression clone resistant to G418 was selected for further experiments. SPC-A-1, H460, GIC-82 and 16-HBE were supplied by Prof. Guang-Biao Zhou [25]. The cells were cultured in Dulbecco's Modified Eagle Medium (DMEM) (Sigma) containing 10% fetal bovine serum (FBS) (Invitrogen), 100 U/ml penicillin, and 100 µg/mL streptomycin at 37°C under 5% CO_2_.

### 3. Cell Viability Assay

#### MTT assay

Cells were plated in 96-well plates at a density of 1×10^4^ cells in 100 µl of medium per well at 24 h before the experiment. Then, cells were then incubated with various concentrations of GNA at 37°C for the indicated duration of time. MTT solution was added to the culture medium (500 µg/ml final concentration) at 4 h before the end of treatment. The reaction was stopped by the addition of 10% acidified SDS (100 µl) to each well. The absorbance value (A) at 490 nm was measured using an Automated Microplate Reader (Bio-Tek ELx 800uv, Bio-Tek Instrument Inc, Winooski, VT, USA). Cell viability was expressed as (Asample - Ablank)/(Acontrol - Ablank)×100%, where A is the absorbance. For the cells treated with reagents, vehicle-treated cells were used as the control. The blank represented MTT added to medium.

#### PI or Annexin V/PI flow cytometry assay

Cells were treated with the indicated concentrations of GNA for 24 hours, harvested by trypsinization, then washed twice with PBS, incubated with PI alone or together with annexin V-fluorescein isothiocyanate for 10 min away from light and evaluated by flow cytometry (Becton & Dickinson, USA). The percentage of dead cells was determined based on the plasma membrane permeability to PI.

#### Trypan blue staining assay

Cells (2×10^5^ cells per well) were seeded in 24-well flat-bottomed plates. After culture for 24 hours, 3 µM GNA was added for the indicated periods of time. The cells were then detached from the substrate by trypsinization and stained with Trypan blue solution (0.4% in PBS). The number of dead cells was counted on a hemocytometer under a light microscope (Olympus, Japan). At least 400 cells were counted for each sample, and the experiment was repeated three times.

### 4. Autophagic marker staining

GFP-LC3/HeLa cells that had been treated with 3 µM of GNA for the indicated periods of time were incubated with 10 µM monodansylcadaverine or 75 nM LysoTracker Red for 15 min. After washing twice with PBS, the cells were examined by fluorescence microscopy (Olympus, Japan).

### 5. Quantification of acidic vesicular organelles with acridine orange

After treatment with the indicated reagents, the cells were stained with acridine orange at a final concentration of 1 µg/ml for 15 min away from light, then observed under a fluorescence microscope or harvested by trypsinization and analyzed by flow cytometry.

### 6. Transmission electron microscopy

Cells were harvested by trypsinization and tumor tissue was resected, then washed twice with PBS and fixed with 2% paraformaldehyde/2% glutaraldehyde in 0.1 M phosphate buffer (pH 7.4), followed by 1% OsO_4_. After dehydration, thin sections were stained with uranyl acetate and lead citrate for observation under a JEOL TEM-100SX electron microscope (JEOL, Japan).

### 7. Immunoblot analysis

Cells or tumor tissue were washed in cold phosphate-buffered saline (PBS) at 4°C, then proteins were prepared, separated by 12% SDS-PAGE and transferred to a polyvinylidene difluoride membrane (Millipore). The membrane was incubated for 1 h in PBS-Tween 20 (0.05%) containing 5% nonfat milk. Primary antibodies were detected using sheep anti-mouse IgG-HRP or anti-rabbit IgG-HRP secondary antibodies (Amersham Pharmacia Biotech). The proteins were visualized using an ECL detection kit (Amersham Pharmacia Biotech). An anti-GAPDH antibody (glyceraldehyde-3-phosphate dehydrogenase; Chemicon) was used to ensure equal protein loading.

To re-probe the membrane with another primary antibody, the membrane was first incubated with Stripping Buffer (50 mM Tris-HCl, pH 6.8, 0.1 mM β-Mercaptoethanol, and 20 mM SDS) for 30 min at 50°C, then subjected to western blot analysis following the standard protocol.

### 8. RNA interference

Double-stranded oligonucleotides targeting 5′-CCACUCUGUGAGGAAUGCACAGAUA-3′ of human Beclin 1 mRNA was synthesized by Shanghai GenePharma (Shanghai, China), and an irrelevant oligonucleotide served as a negative control. The transfection was performed using Lipofectamine 2000 reagent (Invitrogen) according to the manufacturer's instructions. Briefly, the siRNA and Lipofectamine 2000 (Invitrogen) were mixed in Opti-MEM medium (Invitrogen) and incubated for 30 min at room temperature to allow complex formation. Then, the cells were washed with Opti-MEM medium (Invitrogen), and the mixture was added. At 12 h after transfection, the culture medium was replaced with fresh complete medium. The cells were harvested 72 hours after transfection and further analyzed.

### 9. Xenograft mouse model

BALB/cA nude mice (30–40 days old and weighing 18–20 g) were divided into groups containing six mice per group. A549 cells were injected s.c. (2×10^6^ cells per mouse) into the right hind leg of the mice. After the tumors were established (∼50 mm^3^), the mice were i.v. injected with or without 16 mg/kg GNA twice a week for three weeks. At 24 hours after the last i.v. injection, the tumors were isolated for transmission electron microscopy and western blotting analysis.

### 10. Evaluation of pH in lysosomes

After treatment of A549 cells with 3 µM GNA for the indicated periods of time, the cells were incubated with 1 mM LysoSensor Green DND-189 for 15 min. The cells were washed twice with PBS, then examined by fluorescence microscopy (Olympus, Japan).

## Results

### 1. GNA inhibits growth and induces cell death in cancer cells

The effect of GNA on cell growth was investigated using an MTT assay in several human cancer cell lines. We first examined the effect of GNA on the cell viability of A549 and HeLa cells. As shown in Figure 1, GNA inhibited growth in A549 and HeLa cells in a concentration- and time-dependent manner (Figure 1B). To confirm the effects of GNA on lung cancer cells, the MTT assay was repeated in several other lung cancer cell lines (H460, SPA-C-1, Glc-82 and the normal bronchial epithelial cell line 16-HBE). H460, SPA-C-1 and Glc-82 were all sensitive to GNA, whereas 16-HBE was less sensitive to GNA (Figure S1A). These results indicate that GNA can effectively kill lung cancer cells with low toxicity.

Interestingly, when we performed a light microscopy kinetics experiment, we found that the GNA-treated cells showed a progressive accumulation of intracytoplasmic vacuoles that were similar to those often observed in cells undergoing autophagy (Figure 1C). Meanwhile, an increased percentage of cells with large visible vacuoles was observed in the GNA-treated cells compared to the control cells (Figure 1C), consistent with an autophagy-related mechanism.

### 2. GNA increase autophagic markers in A549 and HeLa cells

**Figure 2:**
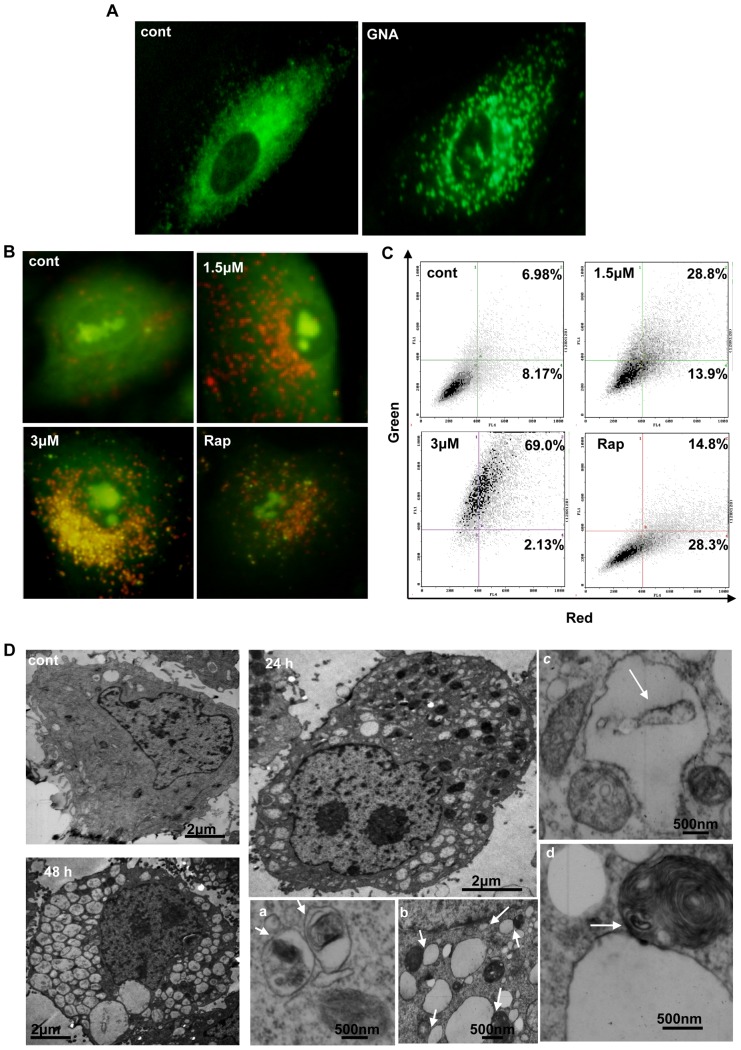
GNA increase autophagic markers in A549 and HeLa cells. A, Representative images of MDC staining. A549 cells were treated with 3 µM GNA for 24 hours, then stained with 10 µM monodansylcadaverine (MDC) and observed under a phase contrast microscope. B and C, Acridine orange staining. Cells were exposed to the indicated concentrations of GNA for 24 hours, then stained with 1 µg/ml of acridine orange (B, C) and observed under a phase contrast microscope (B) or analyzed by flow cytometry (C). Cells that were treated with 50 µg/ml of rapamycin were used as a positive control. The results shown are representative of at least 2 independent experiments. D, Representative electron microscopy images of GNA-treated A549 cells. The cells were treated with 3 µM GNA for the indicated periods of time and analyzed by electron microscopy. Arrows indicate the vacuoles contacting with vesicles that were densely filled with multi-lamellar structures.

A series of experiments were performed to determine whether autophagy is induced by GNA. First, we used monodansylcadaverine (MDC), a lysosomotropic compound known to label acidic endosomes, lysosomes, and autophagosomes [27]. As shown in Figure 2A, GNA-treated A549 cells showed a dramatic increase in the number of MDC-labeled vesicles, indicating that GNA could induce the formation of acidic vesicular organelles (AVO), which is a characteristic of autophagy. To quantify these AVOs, acridine orange vital staining was performed and analyzed by FACS. We found that acridine orange-positive AVOs increased in GNA-treated A549 cells relative to control cells (Figure 2B, 2C); rapamycin was used as a positive control.

The presence of autophagosomes, which display double-membrane vesicle structures, is considered to be the hallmark of autophagy. We examined whether this structure formed in GNA-treated A549 cells under an electron microscope (EM). As shown in Figure 2D, GNA-treated cells displayed increased formation of autophagosomes relative to control cells.

### 3. GNA triggers the formation of autophagic markers in A549 and HeLa cells

**Figure 3:**
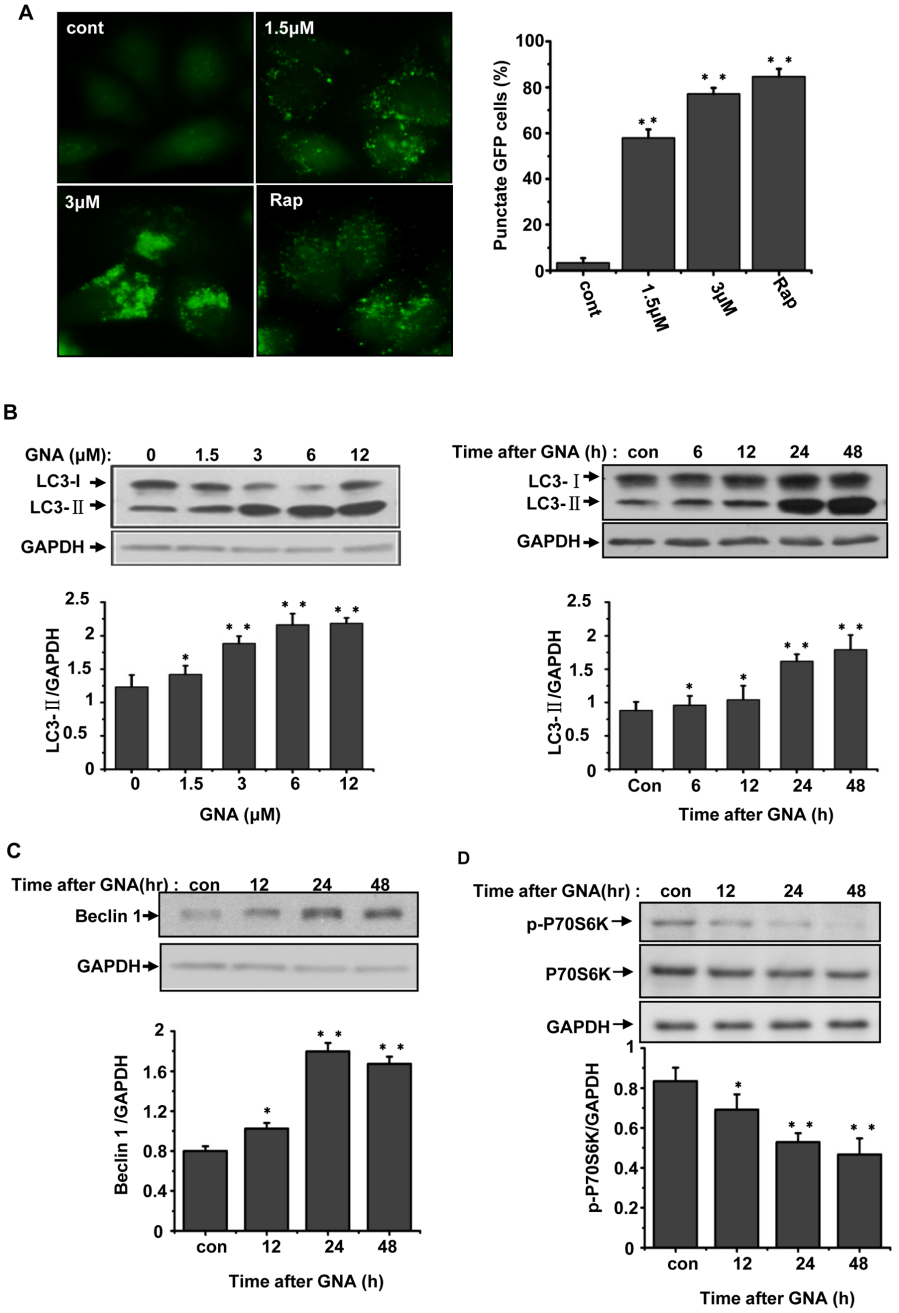
GNA triggers the formation of autophagic markers in A549 and HeLa cell. A, Effects of GNA on the distribution of GFP-LC3 punctuates. GFP-LC3/HeLa cells were treated with various concentrations of GNA or 50 µg/ml of rapamycin for 24 hours, then detected by fluorescence microscopy. The percentage of cells with at least 5 GFP-LC3 punctuate dots was calculated; at least 600 cells were observed, Means ± SEM, n = 3, ** means p<0.01, one-way ANOVA. B, Effects of GNA on LC3 protein. A549 cells were treated with various concentrations of GNA for 24 hours or 3 µM GNA for the indicated periods of time, then analyzed by western blotting using anti-LC3 antibodies. GAPDH protein was used as the loading control. The bar graph shows the band intensities of LC3-II relative to those of GAPDH. Mean± SEM, n = 3, *means p<0.05, ** p<0.01, one-way ANOVA. C, Effects of GNA on Beclin1 protein. A549 cells were treated with 3 µM GNA for the indicated periods of time, then analyzed by western blotting using anti-Beclin1 antibodies. GAPDH protein was used as the loading control. The bar graph shows the band intensities of Beclin 1 relative to those of GAPDH. Mean± SEM, n = 3, *means p<0.05, ** p<0.01, one-way ANOVA. D, Effects of GNA on P70S6K phosphorylation. A549 cells were treated with 3 µM GNA for the indicated periods of time, then analyzed by western blotting using anti-P70S6K and anti-p-P70S6K antibodies. GAPDH protein was used as the loading control. The bar graph shows the band intensities of p-P70S6K relative to those of GAPDH. Mean± SEM, n = 3, *means p<0.05, ** p<0.01, one-way ANOVA.

To confirm the GNA-mediated induction of autophagy, we examined the expression of autophagy markers, including LC3. During autophagy, LC3 is converted from the free form (LC3-1) to a proteolytically processed smaller form (LC3-II). GFP-LC3/HeLa cells, which stably express GFP-LC3, were treated with the indicated concentrations of GNA for 24 hours; these GNA-treated cells exhibited a dramatic increase in the punctuate distribution of GFP-LC3 in a concentration-dependent manner, whereas untreated cells displayed a diffuse GFP-LC3 appearance. Quantitation indicated that the number of cells that contained at least 5 GFP-LC3 punctuate dots also increased in a concentration-dependent manner (Figure 3A). Western blotting analysis of GNA-treated A549 cells showed a remarkable increase in the level of LC3-II in a concentration- and time-dependent manner (Figure 3B). Similar results were obtained in H460, SPA-C-1 and Glc-82 lung cancer cell lines, whereas the normal lung cell line 16-HBE was less sensitive to GNA (Figure S1B).

The levels of Beclin 1, an ATG gene product that is essential for autophagy [28], clearly increased over time in GNA-treated A549 cells (Figure 3C). The ser/thr kinase mTOR acts as one gatekeeper in the autophagy process, and reduced mTOR activity has been associated with elevated levels of autophagy. P70S6K is a substrate of mTOR and its phosphorylation is dependent on mTOR activity. We found that P70S6K phosphorylation clearly decreased over time after GNA treatment (Figure 3D), indicating reduced mTOR activity. Together, these results strongly suggest that GNA triggers the initiation of autophagic markers in A549 and HeLa cells.

### 4. GNA inhibits the fusion between autophagosomes and autolysosomes

**Figure 4:**
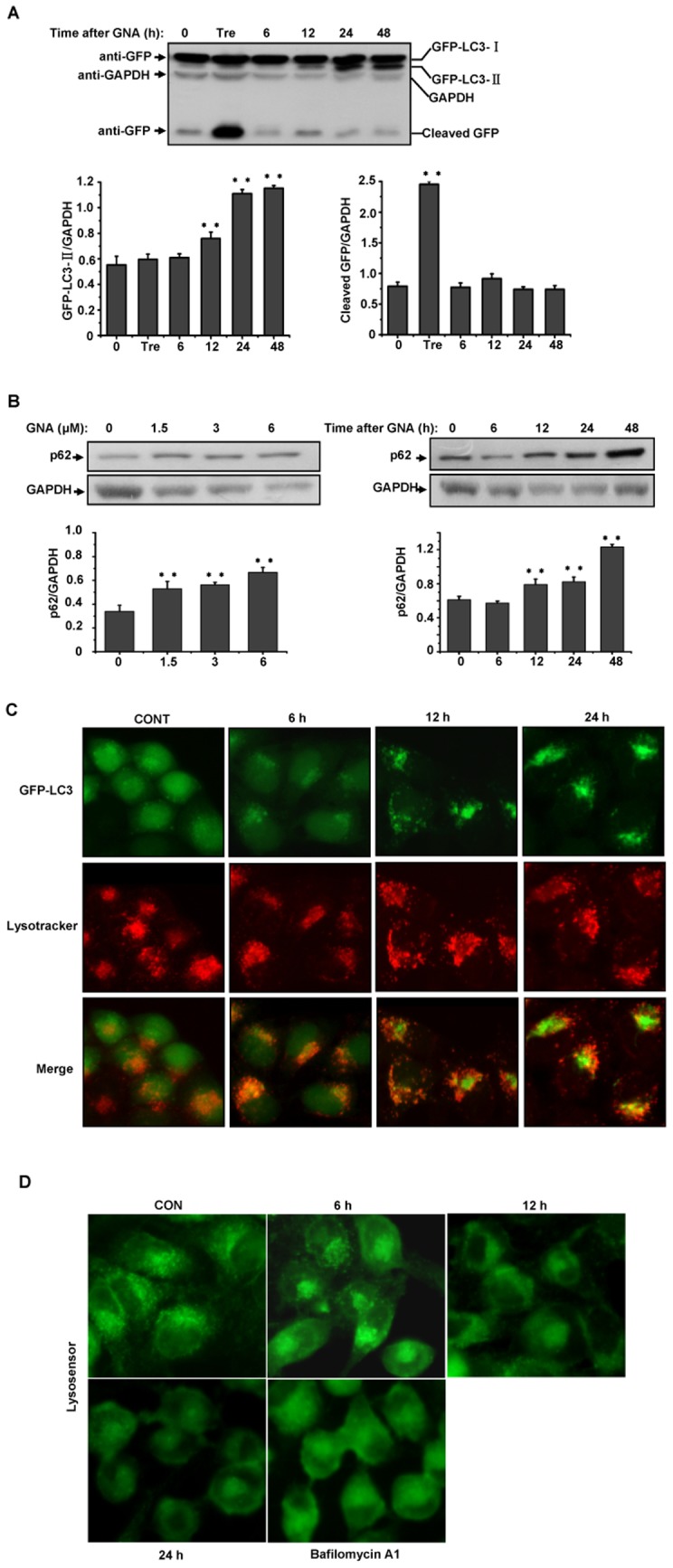
GNA inhibits the fusion between autophagosomes and autolysosomes. A, Effect of GNA on the flux of autophagy. GFP-LC3/HeLa cells were cultured in the absence (control) or presence of 3 µM of GNA for the indicated periods of time, and the level of cleaved GFP was analyzed by western blotting using an anti-GFP antibody; 100 mM trehalose was used as a positive control. GAPDH protein was used as the loading control. The bar graph shows the band intensities of GFP-LC3-II or cleaved GFP relative to those of GAPDH. Mean± SEM, n = 3, *means p<0.05, ** p<0.01, one-way ANOVA. B, Dose- and time-dependent effects of GNA on p62 degradation. A549 cells were treated with various concentrations of GNA for 24 hours or 3 µM GNA for the indicated periods of time, and the p62 protein levels were analyzed by western blotting. GAPDH protein was used as the loading control. The bar graph shows the band intensities of p62 relative to those of GAPDH. Mean± SEM, n = 3, ** p<0.01, one-way ANOVA. C, Inhibition of the fusion between autophagosomes and lysosomes upon treatment with GNA. GFP-LC3/Hela cells were treated with 3 µM GNA for the indicated periods of time and subsequently stained with 75 nM LysoTracker Red (LTR) for 15 min. Representative fluorescence microscopy images are shown. D, Inhibition of lysosome acidification upon treatment with GNA. A549 cells were treated with 3 µM GNA for the indicated periods of time or 500 µM of Bafilomycin A1 for 8 hours and subsequently stained with 1 mM LysoSensor Green DND-189 for 15 min. Representative fluorescence microscopy images are shown.

Because GNA triggered the initiation of autophagic markers, we wondered whether GNA could trigger autophagic flux. To address this question, we assessed whether changes occurred in GFP-LC3, which also has been used to monitor the autophagic flux. When GFP-LC3 is delivered to a lysosome, the LC3 portion of the chimera is sensitive to degradation, whereas the GFP protein is relatively resistant to hydrolysis. Therefore, measuring the levels of cleaved GFP by western blotting can monitor the flux of autophagy. As shown in Figure 4A, the level of free GFP changed slightly upon GNA treatment, while GFP-LC3-II clearly increased over time. Very different results were observed upon treatment with trehalose (a normal autophagy inducer), which was used as a positive control. We further monitored the level of endogenous p62/SQSTM1, an autophagy-lysosome substrate that is associated with autophagy-lysosomal protein degradation. The accumulation of p62 is considered to be a marker for inhibition of autophagy or disruption of autophagic degradation. GNA blocked the degradation of p62 in a concentration- and time-dependent manner (Figure 4B), suggesting that GNA impairs autophagic degradation. Similar results were obtained in H460, SPA-C-1 and Glc-82 lung cancer cell lines, whereas the normal lung cell line 16-HBE was less sensitive to GNA (Figure S1B).

Taken together, these findings strongly suggest that GNA can disrupt the degradation of autophagy at a late stage.

We next questioned which process is affected by GNA. We examined the effect of GNA on the colocalization of autophagosomes and lysosomes. As shown in Figure 4C, numerous small, intensely stained GFP-LC3 dots (autophagosomes) and LysoTracker Red (LTR)-stained dots (lysosomes) were colocalized within 6 hours after GNA treatment. However, this colocalization was disrupted at 12 and 24 hours after GNA treatment; the small, intensely stained GPF-LC3 dots seemed to accumulate and became large, intensely stained regions, which suggested that the fusion between autophagosomes and lysosomes was blocked. Previous studies have shown that some drugs, such as CQ and Bafilomycin A1, can elevate the pH of lysosomes to inhibit fusion with autophagosomes. We questioned whether GNA has similar effects. We assessed the effect of GNA on lysosome acidification by staining with LysoSensor Green DND-189, an acidotropic fluorescent probe that can be trapped in acidic organelles. Bafilomycin A1 was used as a positive control. As shown in Figure 4D, lysosome labeling was disrupted within 12 hours after GNA treatment and was much more obviously affected at 24 and 36 hours (similar to the effects of Bafilomycin A1), indicating inhibition of lysosome acidification. This observation may explain the GNA-mediated inhibition of the fusion between autophagosomes and lysosomes. Therefore, although GNA triggers the formation of autophagosomes, GNA also blocks the progression of the autophagic process by suppressing fusion between the autophagosomes and lysosomes, thereby inhibiting the degradation of the contents.

### 5. The knockdown of Beclin 1 decreases GNA-induced cancer cell death

**Figure 5:**
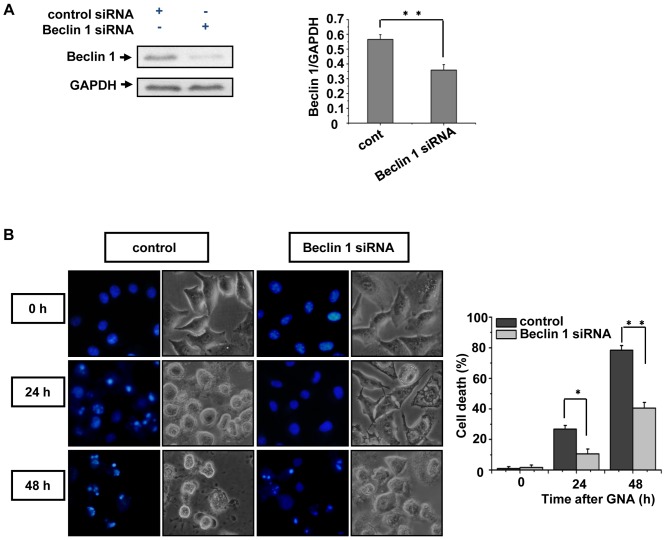
The knockdown of Beclin 1 decreases GNA-induced cancer cell death. A, RNA oligonucleotides against Beclin 1 (Beclin 1 siRNA) effectively repress Beclin 1 expression. A549 cells were transfected with siRNAs against Beclin 1 or negative control, and the protein level of Beclin1 was analyzed by western blotting. GAPDH protein was used as the loading control. The bar graph shows the band intensities of Beclin1 relative to those of GAPDH. Mean± SEM, n = 3, ** p<0.01, one-way ANOVA. B, A549 cells were transfected with siRNAs against Beclin 1 or negative control. The cells were then treated with 3 µM GNA for the indicated periods of time, stained with 2 µM Hoechst 33342 and observed under a fluorescent microscope; the percentage of dead cells was quantified by Trypan blue staining. Mean± SEM, n = 3, *means p<0.05, ** p<0.01, one-way ANOVA.

The previous data indicate that GNA can induce cell death through apoptosis. To determine whether the cell death caused by GNA correlates with dysfunctional autophagy, we further employed small interference RNA (siRNA) to knock down the expression of Beclin 1, an essential gene for autophagy. Transfection of the RNA oligonucleotides against Beclin1 (Beclin 1 siRNA) in A549 cells successfully suppressed the protein level of endogenous Beclin 1 compared with cells transfected with control siRNA, indicating that the Beclin 1 siRNA is efficient (Figure 5A). After 24 or 36 hours treatment with 3 µM GNA, Beclin 1 knockdown cells displayed significantly less cell death compared to control cells (Figure 5B), suggesting that GNA-induced cell death is related with dysfunctional autophagy.

### 6. GNA induces changes in the levels of apoptosis-related proteins

**Figure 6:**
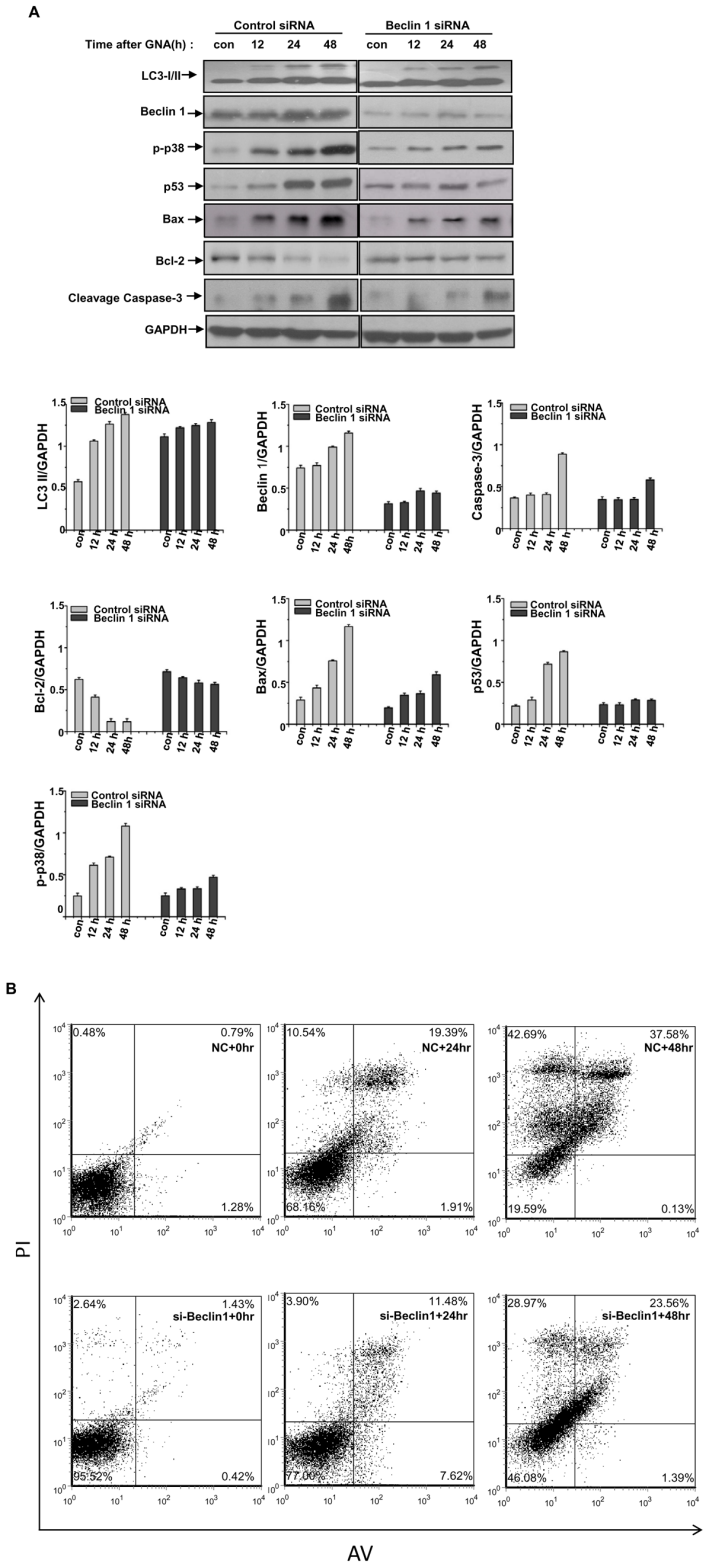
GNA induces apoptosis-related changes in protein levels. A, A549 cells were transfected with siRNAs against Beclin 1 or negative control. The cells were then treated with 3 µM GNA for the indicated periods of time. The relative protein level was analyzed by western blotting. GAPDH protein was used as the loading control. The bar graph shows the band intensities of indicated protein relative to those of GAPDH. Mean± SEM, n = 3, *means p<0.05, ** p<0.01, one-way ANOVA. B, A549 cells were transfected with siRNAs against Beclin 1 or negative control. The cells were then treated with 3 µM GNA for the indicated periods of time, and the cells were stained with annexin V/propidium iodide and subjected to flow cytometry analysis. The percentage in the upper left quadrant indicates the proportion of cells labeled with propidium iodide (dead cells), whereas the percentage in the upper right quadrant indicates the population of cells labeled by both annexin V and propidium iodide (late apoptotic and dead cells).

Next, we investigated the pathway by which dysfunctional autophagy induced cell death occurs upon GNA treatment. Increasing evidence has indicated that the cross-talk between autophagy and apoptosis is especially complicated by the fact that these processes share many common regulatory molecules, such as p53 and Bcl-2 family members [29]–[30]. GNA caused an increase in the protein levels of Bax and cleaved caspase-3 and a decrease in the levels of Bcl-2 and LC3-II over time, suggesting that GNA treatment activates Bax. Because Bax is one of the most prominent downstream targets of p53, we assessed the effect of GNA on p53. As shown in Figure 6A, GNA caused a remarkable increase in the level of p53. Because p38 MAPK contributes to the activation of p53, we also monitored the levels of p38. GNA caused a remarkable increase in the level of phosphorylated p38 (Figure 6A). Furthermore, flow cytometry analysis using annexin V/propidium iodide staining indicated that GNA treatment clearly increased the fraction of dead cells and late apoptotic cells compared with NC. Thus, we conclude that GNA can induce apoptosis in A549 cells at least partially through this pathway.

### 7. GNA inhibits autophagy in vivo

**Figure 7:**
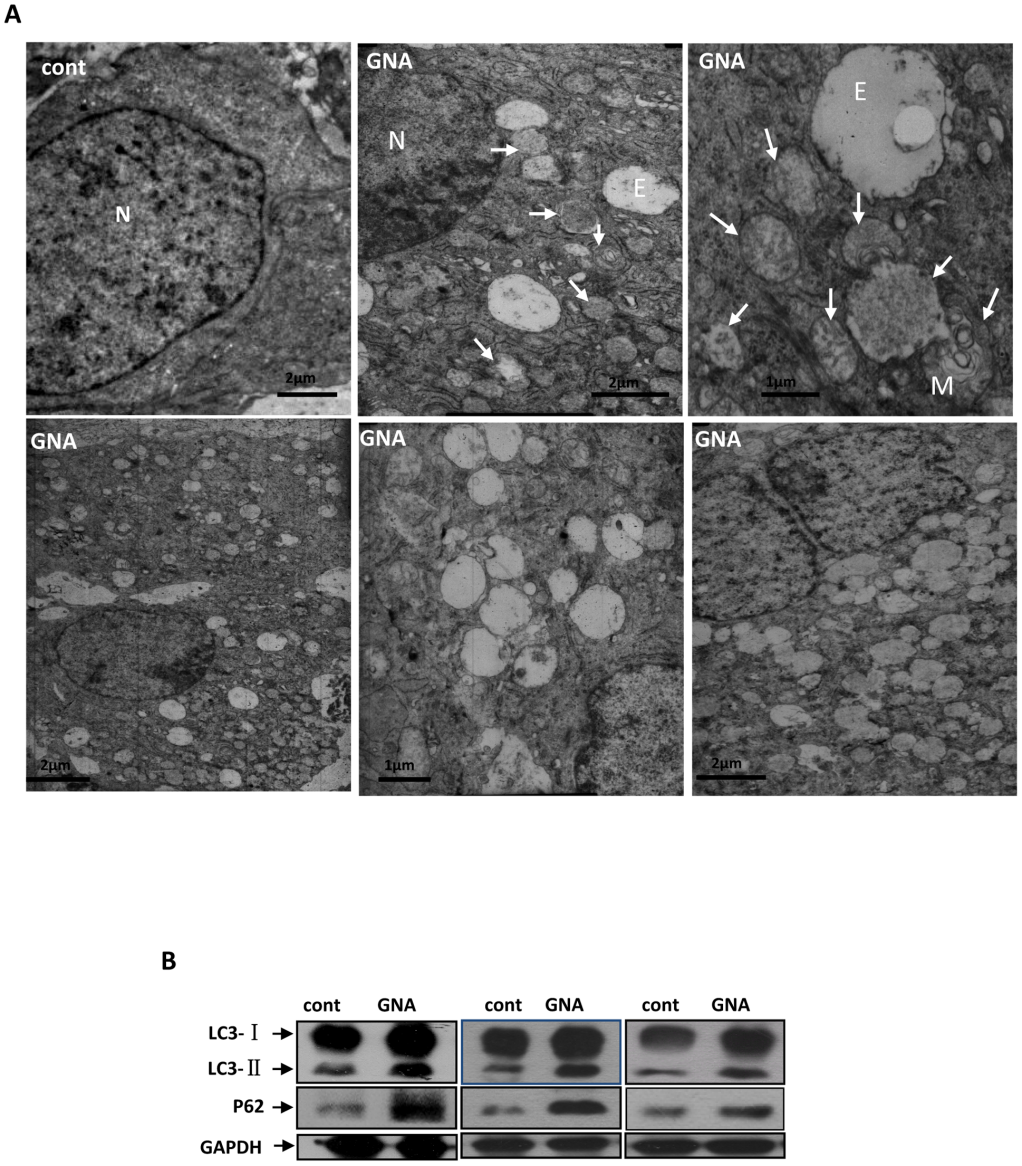
GNA inhibited autophagy in vivo. A549 cells were injected s.c. (2×10^6^/mouse) into 30–40-day-old BALB/cA male nude mice. After established tumors had formed (∼50 mm^3^), the mice were i.v. injected with or without 16 mg/kg GNA twice a week for three weeks. At 24 hours after the last i.v. injection, the mice were sacrificed and the tumors were removed. A, Representative electron microscopy images obtained from the tumors. N, nucleus, E, empty vesicle, M, membranous body. Arrows indicate autophagosomes with contents. B, After preparing tissue homogenate from the tumors, the LC3 and p62 levels were analyzed by western blotting. The results shown are representative of at least 3 independent experiments.

GNA has shown efficacy in tumors growing in nude mice. Our lab has previously shown that the relative tumor volumes (RTV) in mice treated with 16 and 32 mg/kg GNA were 12.16±7.39 and 7.65±2.84, respectively, while the RTV in vehicle-treated negative controls was 26.36±14.10; the relative tumor growth ratios (T/C, %) were 46.1% and 29.0%. To determine the mechanism by which GNA impairs tumor growth *in vivo*, we established a xenograft mouse lung tumor model. As shown in Figure 7, GNA induced the accumulation of autophagic vesicles (Figure 7A), increased the levels of LC3-II (Figure 7B) and blocked p62 degradation (Figure 7B) in tumors. These results strongly suggest that the mechanism by which GNA acts *in vivo* is also autophagy-related.

## Discussion

Recently, many studies have revealed that autophagy is an essential mediator of the effects of many anticancer drugs [6], [31]–[32]. For tumor cells, autophagy acts as both a pro-survival and pro-death mechanism. On the one hand, autophagy maintains cellular metabolism and limits the accumulation of damaged organelles and proteins, which is essential for tumor cell survival. Stress-induced autophagy in tumor cells can lead to treatment resistance and tumor dormancy, with eventual tumor regrowth and progression. Thus, inhibiting autophagy through genetic or pharmacological means induces apoptotic tumor cell death. On the other hand, under extreme stress, sustained activation of autophagy can lead to death in some cancer cells. This situation is called “autophagic cell death,” which is also known as type II programmed cell death. In this paper, we aimed to understand the mechanism by which GNA kills lung cancer cells and the role of autophagy in this process. Our results indicate that GNA can efficiently kill many types of lung cancer cells in an autophagy-dependent manner, and knockdown of the autophagy-related gene Beclin1 abolishes this ability to kill the cancer cells (Figure 6). Thus, autophagy acts as a pro-death mechanism in GNA-treated cells. This toxic autophagic cell death has also been reported by other groups [6], [33]. In GNA-treated cells, the autophagic process was disrupted, reflected by the increased level of p62 and absence of released free-GFP (Figure 4). Further investigation revealed that GNA blocked the fusion between autophagosomes and autolysosomes by inhibiting acidification in the lysosomes. These data led to the questions of whether autophagy-induced cell death is caused by inhibition of autophagy at a late stage. Indeed, some studies have reported that autophagy is presumably activated by dying cells as part of an unsuccessful effort to cope with stress. Preventing the fusion between autophagosomes and lysosomes or reducing lysosomal degradation could lead to a massive accumulation of autophagosomes. In this case, the induction of dysfunctional autophagy would accelerate, rather than prevent, cell death [34]–[38]. Thus, inhibition of autophagy at a late stage by GNA was toxic for tumor cells.

Increasing evidence has indicated that the cross-talk between apoptosis and autophagy is important and that these processes share many common regulatory molecules, such as p53, Bcl-2, Beclin 1 and mTOR signaling pathway members [39]. Inhibition of mTOR pathway signaling causes cell death that is associated with apoptosis and autophagy [40]. In this paper, we demonstrated that GNA regulates mTOR by significantly decreasing the phosphorylation of P70S6K (a substrate of mTOR) over time after GNA treatment (Figure 3D). This process is accompanied by cell death and the activation of Beclin 1, p53 and Bax. The mTOR pathway may contribute to the initiation of autophagosomes and the activation of apoptosis partners after GNA treatment. Our results indicate that inhibition of mTOR by GNA not only induces autophagy but also enhances apoptosis and that excessive autophagy may partner with apoptosis to induce cell death.

Proteins of the Bcl-2 family regulate the apoptosis pathway and autophagy. Bcl-2 associates with pro-apoptotic family members, including Bax, via BH3 domains. The release of Bax from protective Bcl-2 proteins can perturb the mitochondrial membranes, forming pores to release cytochrome c and AIF, which leads to apoptosis. Recently, Bcl-2 has also been shown to inhibit autophagy by antagonizing the BH3-only protein Beclin1, an essential inducer of autophagy. Hence, blocking the Bcl-2-Beclin 1 interaction combined with downregulating Bcl-2 and upregulating Beclin 1 can induce autophagy. Our results showed that the levels of Bcl-2 decreased while Beclin 1 and Bax increased over time in GNA-treated A549 cells. As the levels of Bcl-2 decreased, the Bcl-2-Beclin 1 or Bcl-2-Bax complexes may have been interrupted, releasing Beclin 1 or Bax and inducing autophagy or apoptosis. During this process, Beclin 1 may have contributed to the GNA-induced cell death.

Activation of components of the p38 pathway leads to increased p53 transcriptional activity and induces a transcriptional target of p53 and Bax. In previous studies, our lab and Yu et al. have revealed that GNA causes G0/G1 arrest [22], [25]. In this paper, we demonstrated that GNA activates p38, p53 and Bax while causing a decrease in the level of Bcl-2. Beclin 1 knockdown caused a significant decrease in the expression of LC3-II, p-p38, p53, Bax and caspase-3 but impaired the degradation of Bcl-2. These results suggest that GNA-induced cell death is related to autophagy. However, studies have shown that p38 directs cells to undergo apoptosis or contributes to the further activation of p53, which also contribute to apoptosis [41]. In addition, inhibition of autophagy can also increase apoptosis. Apoptosis may be the result of the activation of p38, p53 and/or the inhibition of autophagy.

Previous studies have revealed that CQ, which elevates the pH of lysosomes to inhibit fusion with autophagosomes, can induce cell death in human colorectal cancer cells dependent upon p53 [42]. Our studies indicated that GNA inhibits the acidification of lysosomes, which suppresses fusion with autophagosomes, thereby inhibiting the degradation of the contents. P53 was also significantly increased during this process. These results indicate that p53 activation plays an important role in GNA-induced cell death.

In conclusion, our study has shown that GNA can block the fusion between autophagosomes and lysosomes by inhibiting acidification in lysosomes. Dysfunctional autophagy played a pro-death role in GNA-mediated cell death. Our data identify a plausible mechanism by which GNA exerts potent anticancer activity and suggest the potential application of GNA as a tool or viable drug in anticancer therapies.

## Supporting Information

Figure S1 **The effects of GNA on SPC-A-1, H460, GIc-82 and 16-HBE cell viability and autophagy.** A, GNA induces growth inhibition in lung cancer cells but not in normal epithelial cells. SPC-A-1, H460, GIc-82 and 16-HBE cells were treated with various concentrations of GNA for the indicated periods of time, and cell proliferation was analyzed by the MTT assay. The data are expressed as the means of 4 independent experiments performed at least in duplicate. The error bar represents the S.E. B, Effects of GNA on LC3 protein. SPC-A-1, H460, GIc-82 and 16-HBE cells were treated with 3 µM GNA for the indicated periods of time, then analyzed by western blotting using anti-LC3 and p62 antibodies. GAPDH protein was used as the loading control.
(TIF)
