# Supplementary figures and images for: Gambogenic Acid Kills Lung Cancer Cells through Aberrant Autophagy
Source: PLoS One. 2014 Jan 10;9(1):e83604. doi: 10.1371/journal.pone.0083604 (PMC3888381; doi:10.1371/journal.pone.0083604)

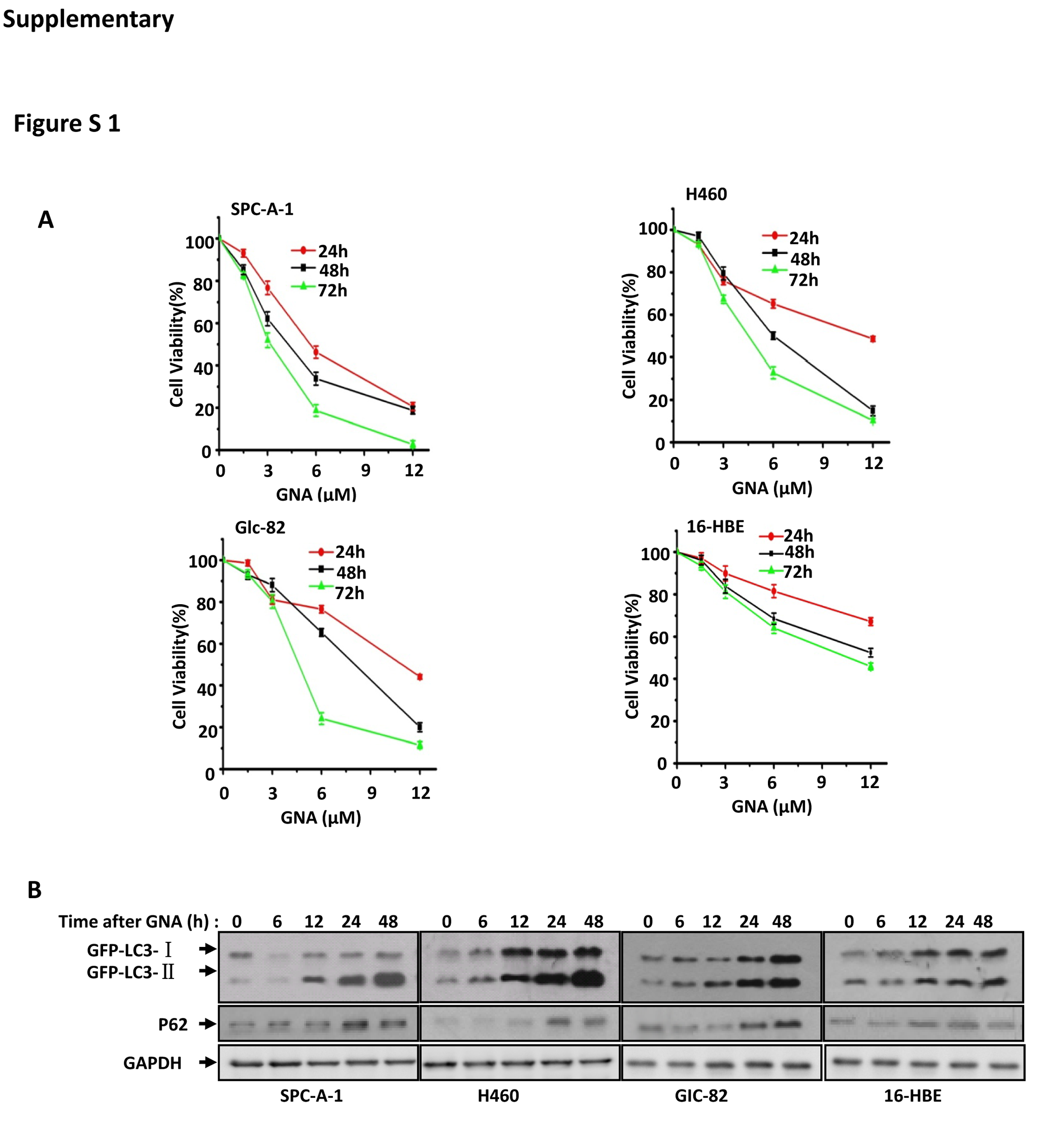

Supplement: Figure S1 — The effects of GNA on SPC-A-1, H460, GIc-82 and 16-HBE cell viability and autophagy. A, GNA induces growth inhibition in lung cancer cells but not in normal epithelial cells. SPC-A-1, H460, GIc-82 and 16-HBE cells were treated with various concentrations of GNA for the indicated periods of time, and cell proliferation was analyzed by the MTT assay. The data are expressed as the means of 4 independent experiments performed at least in duplicate. The error bar represents the S.E. B, Effects of GNA on LC3 protein. SPC-A-1, H460, GIc-82 and 16-HBE cells were treated with 3 µM GNA for the indicated periods of time, then analyzed by western blotting using anti-LC3 and p62 antibodies. GAPDH protein was used as the loading control. (TIF) [file pone.0083604.s001.tif]
